# Targeting Dendritic Cells with Virus-like Particles: Toward Safer and More Immunogenic Vaccines

**DOI:** 10.3390/vaccines13111142

**Published:** 2025-11-06

**Authors:** Jonny Jonny, Chairul A. Nidom, Terawan A. Putranto, Soetojo Wirjopranoto, I Ketut Sudiana, Elisa D. Pratiwi, Tiza W. Mawaddah, Astria N. Nidom, Reviany V. Nidom, Setyarina Indrasari, Irma Y. Rosytania, Astrid D. Larasati

**Affiliations:** 1 Doctoral Program of Medical Science, Faculty of Medicine, Airlangga University, Surabaya 60132, Indonesia; soetojo@fk.unair.ac.id (S.W.); i-ketut-s@fk.unair.ac.id (I.K.S.); astriann@pnfinstitute.org (A.N.N.); 2 Indonesia Army Cellcure Center, Gatot Soebroto Central Army Hospital, Jakarta 10410, Indonesia; terawanagusputranto@unprimdn.ac.id (T.A.P.); astrid.devinalarasati@gmail.com (A.D.L.); 3 Faculty of Military Medicine, Indonesia Defense University, Bogor 16810, Indonesia; 4 Faculty of Veterinary Medicine, Airlangga University, Surabaya 60115, Indonesia; elisadiahpratiwi@pnfinstitute.org (E.D.P.); tizawurimawaddah@pnfinstitute.org (T.W.M.); 5 Professor Nidom Foundation, Surabaya 60236, Indonesia; reviany@pnfinstitute.org (R.V.N.); setyarina_ire@pnfinstitute.org (S.I.); irma.yuritaa29@gmail.com (I.Y.R.); 6 Global BiosainsTeknologi, Malang 65145, Indonesia

**Keywords:** dengue virus-like particles (VLPs), dendritic cell immunotherapy, tetravalent dengue vaccine, preclinical toxicology, antigen-based immunotherapy, immune vaccine, BALB/c Mice

## Abstract

**Background/Objectives**: The dengue virus remains endemic in over 100 countries, transmitted by mosquito bites. Current management relies on supportive care, as no highly effective vaccine or approved antiviral exists. The CYD-TDV (Dengvaxia^®^) vaccine, licensed since 2015 with around 60% efficacy, raises the risk of severe dengue in seronegative children. The newer “Qdenga” vaccine offers up to 80% efficacy after a year but provides suboptimal protection against DENV-3 in seronegative individuals. Over the past two decades, virus-like particles (VLPs) have gained attention as safe, replication-incompetent vaccine platforms. This study evaluates the toxicity profile of dengue VLP-based antigens in BALB/c mice. **Methods**: A total of 80 BALB/C mice were randomly divided into two experimental groups: acute and chronic. Each group consisted of a treatment subgroup (10 males and 10 females) and a control subgroup (10 males and 10 females). In the acute group, the VLP was administered intramuscularly on day 1, while in the chronic group, a second VLP dose was given on day 14. The study was conducted over a 28-day period. Throughout the experiment, body temperature, body weight, mortality, and clinical signs were monitored regularly to assess the functional condition of various organs. **Results**: The results showed no notable alterations in mortality rates, body temperature, body weight, clinical signs, or histopathological observations of the examined organs across all groups, including in the hematological and blood biochemical parameters. **Conclusions**: The administration of tetravalent dengue VLP vaccine in BALB/c mice did not result in adverse effects in acute or chronic toxicity evaluations. Therefore, the VLP supports progression toward clinical evaluation, with dendritic cell activation providing additional rationale.

## 1. Introduction

Dengue fever is a major global health concern, with reported cases rising from 505,430 in 2000 to an estimated 7.6 million in 2024 [1]. This viral disease is vectored by the bites of Aedes albopictus and Aedes aegypti mosquitoes, which are widespread in over 100 countries and are responsible for infecting nearly 400 million individuals each year [2]. The dengue virus (DENV) is an enveloped RNA virus with a single-stranded, positive-sense genome. It belongs to the *Flaviviridae* family under the *Flavivirus* genus and is categorized into four antigenically unique serotypes: DENV-1 through DENV-4 [3].

In the coming decades, climate change is expected to expand the habitats of vector hosts, leading to a greater number of people at risk of dengue infection [4]. Clinical symptoms of dengue can vary, ranging from asymptomatic or similar to flu, to more severe conditions that can lead to a mortality rate of more than 20% if not treated properly [5]. The infection is manifested by fever, headache, joint pain, and muscle pain. In severe cases, the disease can advance to Dengue Hemorrhagic Fever or Dengue Shock Syndrome (DHF/DSS) [6].

Infection by a single dengue virus serotype induces serotype-specific immunity, the duration of which remains under debate, while it confers only temporary and partial cross-protection against heterologous serotypes [7]. At present, no therapeutic drugs have been authorized for dengue treatment, underscoring the critical need for an efficacious vaccine to address this worldwide public health issue.

Efforts to develop dengue vaccines are intricately linked to the challenge posed by Antibody-Dependent Enhancement (ADE), a mechanism that may intensify disease severity during subsequent infections [8]. Although clear clinical evidence is still lacking, the potential risk of ADE remains, highlighting the need for a dengue vaccine to generate robust Neutralizing antibodies (Nab) targeting all four serotypes simultaneously [2]. This concept underpins the development of a tetravalent dengue vaccine designed to elicit concurrent immune responses against all four DENV serotypes: DENV-1, DENV-2, DENV-3, and DENV-4.

The tetravalent dengue vaccine CYD-TDV, previously evaluated in clinical trials, raised safety concerns due to reports indicating increased severity of dengue infection noted in seronegative populations following natural infection post-vaccination [9]. Among the dengue vaccines currently authorized for use are Dengvaxia and Qdenga (TAK-003). Dengvaxia, licensed in 2016, poses a higher risk of severe dengue in seronegative recipients, particularly in children and adolescents, and is therefore recommended only for individuals with confirmed prior dengue infection [9]. Qdenga lacks sufficient data to assess potential disease enhancement in dengue-naïve children, especially after DENV3 and DENV4 infection [2].

VLPs are an alternative vaccine that is more immunogenic than other subunit vaccines. They display repeated surface antigenic epitopes in a conformation that closely resembles the native virus, allowing efficient recognition by the immune system [10]. VLPs are generated when viral structural proteins undergo self-assembly, mimicking the architecture of native virions that lack viral genetic material. The use of attenuated dengue vaccines remains an important strategy, and they are licensed in defined populations; however, they carry inherent risks such as uncontrolled replication or reversion. VLP-based vaccines, being non-replicating, offer an alternative with a favorable safety profile [11].

VLPs are safe for immunocompromised people as they do not contain the genetic material required for virus replication [2]. Studies with mouse models show that dengue VLPs elicit responses from cytotoxic T cells [12], induce anti-DENV antibodies [13], and do not appear to induce ADE in a subsequent DENV infection [14]. In addition to vaccine effectiveness, there is another important focus on safety in vaccine testing through vaccine toxicity. Therefore, this study aimed to evaluate the toxicity of dengue virus-like particles (VLPs) in mice.

In addition to their safety advantages, virus-like particles (VLPs) have been widely reported to stimulate dendritic cells, promoting their maturation and enhancing antigen presentation. Dendritic cells play a pivotal role in initiating adaptive immune responses by processing and presenting antigens to T cells. Several studies have demonstrated that dengue and *flavivirus* VLPs can effectively activate dendritic cells both in vitro and in vivo, positioning them as attractive candidates for immunotherapy strategies [10].

BALB/c mice were chosen because they are a well-established model for vaccine and toxicology studies, with immune responses that are well characterized and comparable across studies. Using both sexes allowed assessment of sex-related variability. This model is relevant to human biology as it provides predictive information on vaccine safety and immune activation prior to clinical trials [13]. In this context, the current study evaluates the preclinical toxicity profile of tetravalent dengue VLPs in BALB/c mice, providing a foundation for future investigations on their immunogenicity and potential in dendritic cell-based immunotherapy.

## 2. Materials and Methods

### 2.1. Ethical Approval

All animal procedures in this study were conducted in accordance with relevant national laws and institutional guidelines. All experiments were performed in an Animal Biosafety Level-3 (ABSL-3) Laboratory at the Professor Nidom Foundation (PNF Facilities). The study protocol was reviewed and approved by the Institutional Animal Care and Use Committee (IACUC) of Professor Nidom Foundation under approval number 010724/IACUC/VII/2024, dated July 2024. This study involved no human participants.

### 2.2. Animals

The experiments used eighty male and female BALB/c mice (5–6 weeks old) obtained from the Animal Division of Biofarma Laboratories, Indonesia. Healthy, SPF BALB/c mice (5–6 weeks old) were included. No A Priori exclusion criteria were set, and all animals/data points were analyzed. All animal care and experimental procedures were conducted in accordance with the institutional guidelines for the care and use of laboratory animals established by the Professor Nidom Foundation. The study was conducted in an Animal Biosafety Level-3 (ABSL-3) Laboratory at the Professor Nidom Foundation (PNF Facilities).

The animals were housed separately according to different treatments. The animals were housed in an environment where temperature (22 ± 2 °C) and lighting (12 h light/dark cycle) were regulated, and food and water were supplied Ad Libitum. All animals and data points were included in the analysis, with no exclusions made. No unexpected adverse events occurred; all animals remained healthy until study completion. Upon completion of the study, all animals were humanely euthanized using a ketamine dosage of 100 mg/kg body weight and xylazine (4 mg/kg body weight) injected subcutaneously for analgesia [15]. Blood and tissue specimens were obtained during necropsy for hematological, biochemical, and histopathological examination.

### 2.3. Vaccination Schedule

A total of 80 male and female BALB/c mice were randomly distributed into two groups for experimentation, representing acute and chronic toxicity studies. Group size n = 80 was based on previous preclinical vaccine safety studies in BALB/c mice and the 3R principles. No formal power calculation was performed. We used 80 male and female BALB/c mice, which were divided into two groups: the acute toxicity group (40 mice) and the chronic toxicity group (40 mice). Each toxicity group was further divided by sex (20 females and 20 males). Within each sex, animals were allocated to the vaccinated group (10 mice) and the control group (10 mice). Animals were randomized within each toxicity group and sex using a computer-generated random sequence (1:1 allocation) to vaccinated or control groups. The randomization list was prepared before allocation. To minimize potential confounders, the order of treatments and sample collections was alternated between groups, and cages were distributed randomly within the animal facility.

The acute group was divided into male and female subgroups, where each subgroup had the vaccinated and the control population, with ten mice in each population. The vaccinated animals in the acute group received a total dose of 0.1 mL (0.05 mL into each thigh muscle) of the dengue VLP, corresponding to a concentration of 0.1 µg/mL, while the control animals received the same total volume of PBS (Phosphate-Buffered Saline), administered intramuscularly on Day 1 without any adjuvant.

The chronic group consisted of male and female subgroups, with the vaccinated and the control population consisting of ten mice in each population. Chronic group animals were injected twice on day one (Day 1) and day 14 (Day 14), both with a total dose of 0.1 mL (0.05 mL into each thigh muscle) of dengue VLP for the vaccinated population, or PBS for the control population, administered intramuscularly on Day 1 and Day 14, without adjuvant.

Group allocation was known to the investigators during animal allocation and the conduct of the experiment. However, outcome assessment and data analysis were performed using coded samples to reduce bias.

### 2.4. Clinical Observations

The physical parameters included condition, mucosa color, lacrimation, salivation, convulsion, tremors, biting signs, piloerection, respiratory, eyelid, and gait were observed. This was an exploratory toxicology study without an a priori sample size calculation; all clinical, hematological, biochemical, and histopathological outcomes were assessed descriptively.

### 2.5. Toxicology Studies

This preclinical toxicology study followed the WHO Guidelines on non-clinical evaluation of vaccines (WHO TRS, no.927, 2005, Annex 1). The duration of this preclinical study was 28 days in which the mortality, clinical signs, body temperature, body weight, hematology, blood biochemistry, and histopathology test of the mice were examined to assess the functional performance of different organs.

### 2.6. Hematological and Biochemical Analysis

Whole blood was collected from the mice Via intracardiac in blood collection tubes after they were euthanized. Venous blood was analyzed to determine the Complete Blood Count (CBC). The plasma of the blood was analyzed for the levels of creatinine, Blood Urea Nitrogen (BUN), total protein, globulin, albumin, albumin/globulin ratio, total glucose, Serum Glutamic Pyruvic Transaminase (SGPT), and Serum Glutamic Oxaloacetic Transaminase (SGOT). Blood samples for the acute group were collected on day 14, while blood samples for the chronic group were collected on day 28.

### 2.7. Histopathological Analysis

We collected brain, bone marrow, heart, liver, lungs, thymus, spleen, kidneys, lymphoid tissue, reproductive organs, and muscle tissues for histopathological analysis. These tissues were stained using Hematoxylin and Eosin (H&E) staining. In brief, small fragments of fresh tissue were fixed in 10% buffered formalin, then gradually dehydrated, embedded in paraffin, sectioned at 5 µm thickness, deparaffinized with p-xylene, rehydrated through graded ethanol, rinsed in water, and finally stained with Hematoxylin and Eosin (H&E) [16]. The histopathological slides were examined by a pathologist using a light microscope with 400× magnification. Tissue damage classification was performed using a modified standard specifically developed for this study. Cells/tissues are said to be normal if there is less than 20% congestion, hemorrhage less than 20%, necrosis less than 5% and degeneration less than 15%, but if otherwise or part of the organ has a value more than the provisions, then the organ is assessed as not normal.

### 2.8. Statistical Analysis

Data for the analysis are presented as mean ± Standard Error (SE). The data obtained from temperature profile, body weight, hematology, and blood chemistry parameters were statistically analyzed using SPSS with ANOVA (Analysis of Variance). When significant differences were observed (*p* < 0.05), Duncan’s test was applied. Histopathological examinations were evaluated descriptively by calculating the percentage of organs showing alterations in each treatment group. Statistical significance was defined as a *p*-value below 0.05 when comparing the control and treatment groups. Statistical analysis was conducted using SPSS version 24.

## 3. Results

### 3.1. Body Temperature

**Figure 1:**
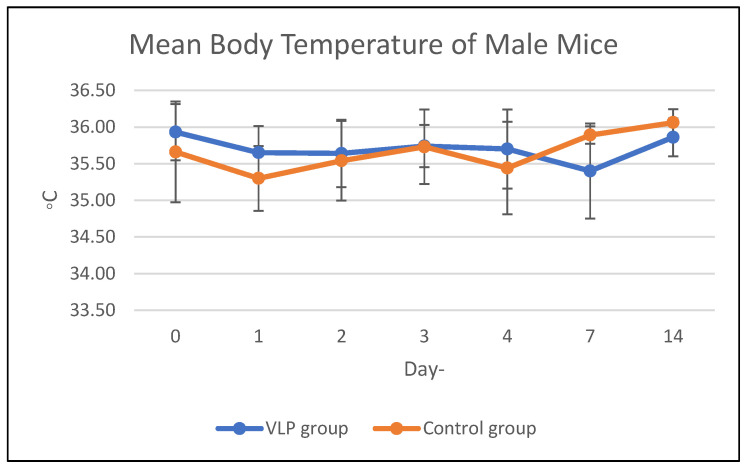

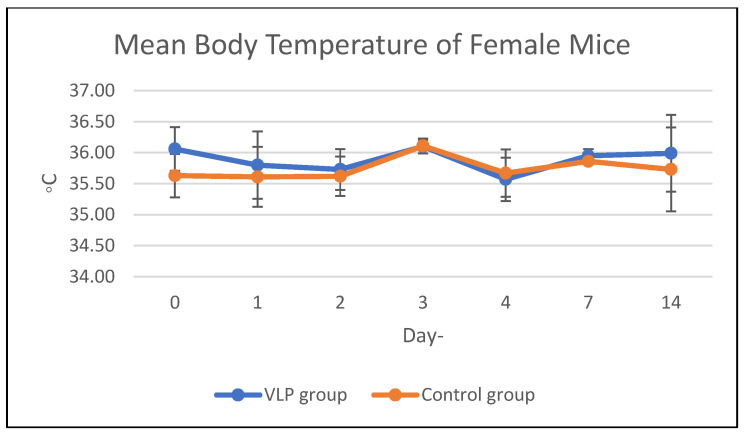
Mean body temperature of mice in the acute group. Mean body temperature (°C) of male and female BALB/c mice in the acute toxicity study following dengue VLP or control (PBS) administration at different observation days.

**Figure 2:**
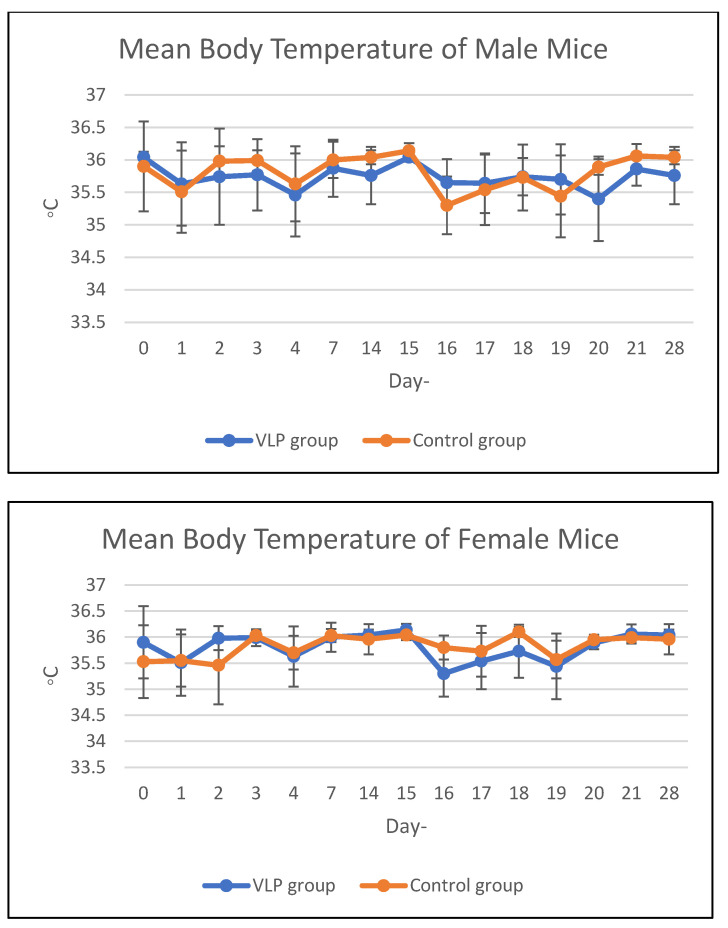
Mean body temperature of mice in the chronic group. Mean body temperature (°C) of male and female BALB/c mice in the chronic toxicity study following dengue VLP or control (PBS) administration at different observation days.

Body temperature in the acute and chronic vaccine groups remained within the same range as that of the control group during the study period (Figure 1 and Figure 2). Details data are provided in the Supplementary Materials (Tables S1 and S2).

### 3.2. Body Weight and Food Consumption

**Figure 3:**
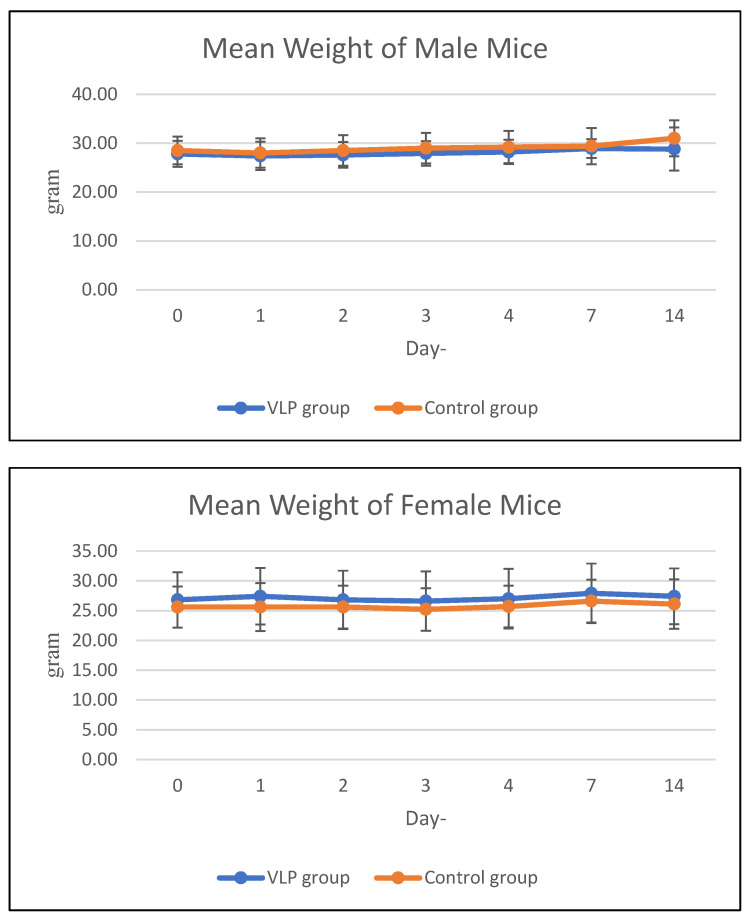
Mean body weight of mice in the acute group. Mean body weight (g) of male and female BALB/c mice in the acute toxicity study at various observation days after dengue VLP or control (PBS) administration.

**Figure 4:**
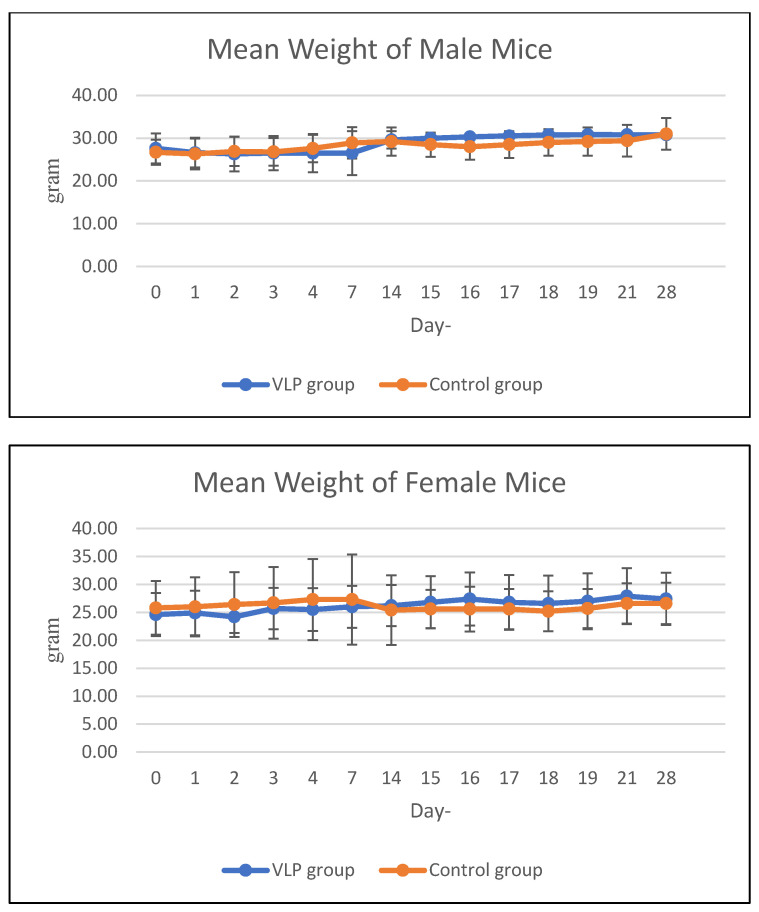
Mean body weight of mice in the chronic group. Mean body weight (g) of male and female BALB/c mice in the chronic toxicity study at various observation days after dengue VLP or control (PBS) administration.

The average weekly feed consumption of mice increased in the first week, then decreased in the 2nd week in all mice in the acute treatment group (both males and females). In the chronic group, there was a decrease in feed consumption in the 2nd week and 3rd week, then an increase again in the 4th week. The average body weight of animals treated with acute and chronic groups was in the same range as the control during the study period (Figure 3 and Figure 4). Details data are provided in the Supplementary Materials (Tables S3 and S4).

### 3.3. Clinical Observations

We did not find any deaths of the mice during the study. None of the mice showed clinical symptoms that indicated illness. The physical parameters were also within a normal range, including condition, mucosa color, lacrimation, salivation, convulsion, tremors, biting signs, piloerection, respiratory, eyelid, and gait.

### 3.4. Hematological

The CBC analysis indicated no significant alterations (*p* > 0.05) in all CBC parameters (Table 1 and Table 2).

### 3.5. Biochemical Analysis

The results of the statistical analysis showed that several blood chemistry parameters were not significantly different (*p* > 0.05) in both the acute and chronic groups (Table 3 and Table 4).

### 3.6. Organ Weight

Based on the mice organ weight data, it showed that all variables given treatment had the same organ weight range as the control.

### 3.7. Effect of VLP Dengue Vaccine on Clinical Pathology and Histopathology Changes

**Figure 5:**
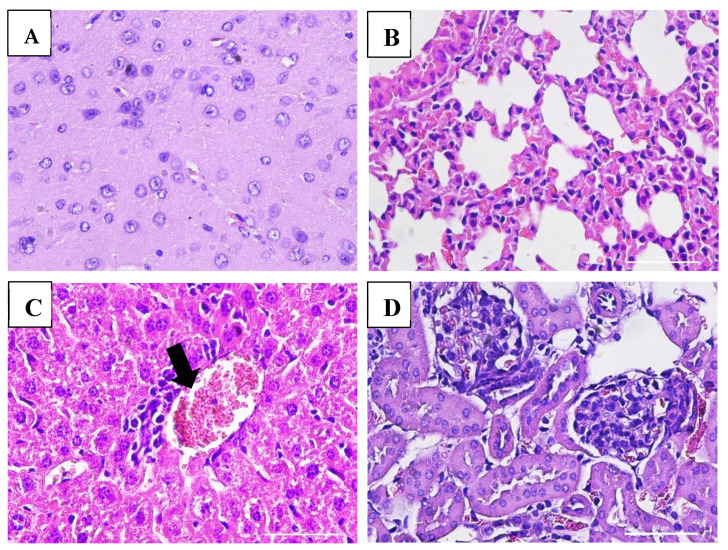
Histopathological images of organs from female mice in the acute VLP treatment group stained with Hematoxylin and Eosin (H&E), at 400× magnification: (**A**) Brain (control group): no pathological changes observed; (**B**) Lungs (VLP group): no pathological changes observed; (**C**) Liver (VLP group): mild inflammatory cell infiltration; (**D**) Kidney (VLP group): no pathological changes observed.

**Figure 6:**
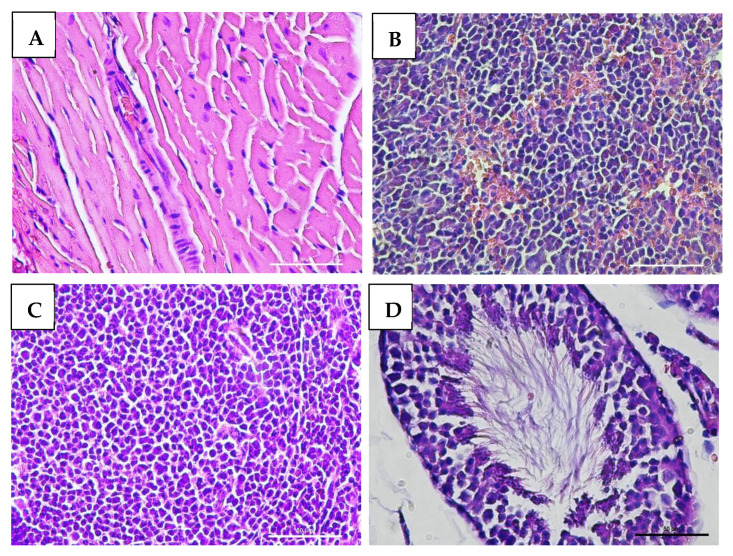
Histopathological images of organs from male mice in the acute VLP treatment group, stained with Hematoxylin and Eosin (H&E), at 400× magnification: (**A**) Heart (control group): normal histological appearance; (**B**) Spleen (VLP group): no histopathological alterations observed; (**C**) Lymph node (VLP group): no histopathological alterations observed; (**D**) Testis (VLP group): normal seminiferous tubule morphology.

**Figure 7:**
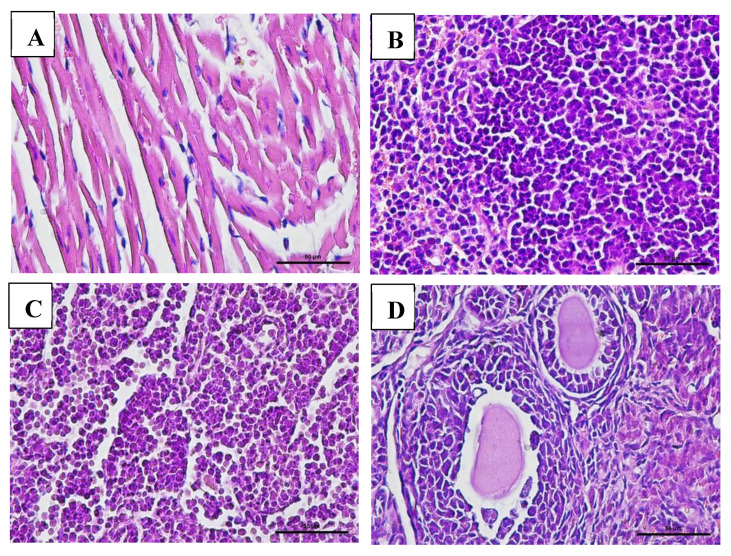
Histopathological images of organs from female mice in the chronic VLP treatment group, stained with Hematoxylin and Eosin (H&E), at 400× magnification: (**A**) Heart (VLP group): no histopathological abnormalities observed; (**B**) Spleen (VLP group): no histopathological abnormalities observed; (**C**) Lymph node (VLP group): normal lymphoid structure; (**D**) Ovary (control group): normal follicular morphology.

**Figure 8:**
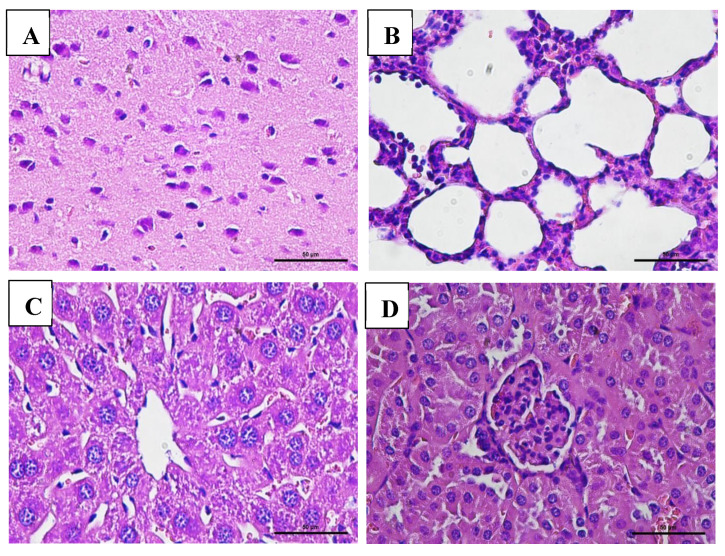
Histopathological images of organs from male mice in the chronic VLP treatment group, stained with Hematoxylin and Eosin (H&E), at 400× magnification: (**A**) Brain (VLP group): normal histological structure; (**B**) Lungs (VLP group): no pathological alterations observed; (**C**) Liver (VLP group): no pathological alterations observed; (**D**) Kidney (control group): no pathological alterations observed.

Macroscopic examination of the organs showed no changes or lesions in either the acute or chronic groups as well as the control group. Clinical pathology and histopathological evaluations of the brain, bone marrow, heart, liver, lungs, thymus, spleen, kidneys, lymphoid tissues, reproductive organs, and muscle tissue in both male and female subjects from the treatment and control groups revealed findings of congestion (<20%), hemorrhage (<20%), necrosis (<5%), and degeneration (<15%) (Figure 5, Figure 6, Figure 7 and Figure 8).

## 4. Discussion

Before conducting clinical trials of vaccines with human volunteers, it is necessary to conduct preclinical evaluation of vaccines using experimental animals as research models. Animals used in this test are expected to show the same immune response in humans after vaccination. These preclinical toxicity studies are designed to demonstrate the safety and tolerability of vaccine product candidates. This study aims to evaluate the safety of a tetravalent dengue VLP vaccine. The dengue virus consists of four separate serotypes: DENV-1, DENV-2, DENV-3, and DENV-4, and these four serotypes have different variations in severity. Some studies state that severe cases often occur with DENV-3 and DENV 1–4 [17]. The challenge in making a dengue vaccine is that the vaccine must be effective in groups at high risk of developing immunity to all four dengue serotypes, as well as providing protection over a long period of time.

The tetravalent vaccine aims to offer lasting immunity against all four serotypes and is expected to reduce the risk of dengue vaccine ADEs. The tests included acute toxicity for 14 days and chronic toxicity for 28 days with the BALB/c mouse animal model to evaluate the safety of tetravalent dengue VLP antigens. Preclinical toxicology studies included the assessment of clinical symptoms, body weight, feed consumption, body temperature, organ weight, hematology, blood biochemistry, and histopathology.

The body weight observation of BALB/c mice revealed no considerable difference between the acute and chronic groups compared to the control group. Normal body temperature of a mouse ranges from 36.5 ± 1.3 °C [18]. According to another study, the average rectal temperature of mice ranges from approximately 36 °C in a cold environment (around 15 °C) to 38 °C in a hot environment (around 35 °C). These findings suggest that mice maintain thermoregulation across a broad range of ambient temperatures [19]. The results of body temperature measurements in mice indicate that most subjects exhibited subnormal temperatures, which may have been influenced by the cold housing conditions.

Hematological and blood biochemical observations serve as fundamental references for the interpretation of diseases and the diagnosis of tissue or organ damage. Hematological and biochemical profiles in mice exhibit variability that can be attributed to factors such as genetic background, strain, and sex, as well as external influences including age, nutritional status, environmental conditions, sampling location, and additional biological or environmental variables [20].

The data revealed that hematocrit levels in the control group mice were within the normal range, based on reference values for BALB/c mice: 39.81 ± 1.64% for males and 40.74 ± 1.71% for females [21]. In the treatment group, the lowest hematocrit levels were observed in the chronic group; however, statistical analysis revealed no significant differences (*p* > 0.05) compared to the control group in either male or female mice. Several factors may contribute to decreased hematocrit levels, one of which is red blood cell damage [22]. Additionally, improper blood collection techniques, such as using microhematocrit tubes that are exposed to the external environment before being transferred to Ethylenediaminetetraacetic Acid (EDTA) tubes, may cause hemolysis and subsequently affect hematocrit measurements [23]. However, in the chronically vaccinated group, hematocrit levels did not differ significantly from the normal range. Since both groups received vaccines with the same composition, it is likely that the difference observed in the acute group was not due to the vaccine formulation itself.

The removal of aged erythrocytes from circulation is primarily mediated by macrophages in the spleen and bone marrow, which contributes to a reduction in hematocrit levels [24]. The decrease in hematocrit is consistent with the decline in hemoglobin and erythrocyte counts. Hemoglobin plays a crucial role in electron transport, as well as in the reduction and transfer of oxygen for hydroxylation reactions [25]. The results indicated that the acute vaccine group of male mice exhibited the lowest hemoglobin and erythrocyte values. In mice, hemoglobin levels typically range from 10 to 17 g/dL [25]. Based on these reference values, the average hemoglobin levels in all groups of mice remained within the normal range. The average normal Red Blood Cell (RBC) count in BALB/c mice is approximately 9 ± 0.44 × 10^6^/µL [21]. Although the observed values differed slightly from the reference, particularly in the chronic group, comparisons with the control group showed no significant differences. Therefore, RBC values in all treatment groups were considered to be within the normal range.

The normal range of white Blood Cell Count (WBC) in mice is reported to be between 2–10 × 10^3^/µL [26,27]. In the present study, WBC in both the acute and chronic vaccination groups remained within this reference range. Additionally, statistical comparison with the control group showed no significant differences, further supporting this observation. Platelet activation in mice can occur spontaneously and is known to be strain-dependent [26]. Platelets are derived from megakaryocytes in the bone marrow and spleen, with thrombopoietin—primarily produced in the liver—serving as the main growth factor [27]. The lifespan of platelets in mice is approximately 5 days, which is shorter than in many other species [26]. In this study, no significant differences in platelet counts were observed between the control and treatment groups.

Urea, creatinine, and albumin levels are commonly used as indicators in kidney function assessment. Normal Blood Urea Nitrogen (BUN) levels in mice range from 21 to 26 mg/dL, while creatinine levels range from 0.2 to 0.9 mg/dL [28]. A reduction in blood urea levels may serve as a clinical sign of impaired hepatic function, as the liver is responsible for converting ammonia to urea during the urea cycle. In this study, BUN levels in all groups were outside the normal reference range; therefore, comparisons were made against the control group under the same treatment conditions. This deviation may be influenced by differences in the maintenance environment.

The normal mean albumin levels in male BALB/c mice are reported to be 2.4 ± 0.47 g/dL and in females 1.74 ± 0.50 g/dL [21]. According to the reference, the average albumin levels observed in this study were within the normal range, and no significant differences were found between the treatment groups. Similarly, the normal mean total protein levels in male BALB/c mice are 5.21 ± 0.46 g/dL and in females 5.23 ± 1.68 g/dL [21]. The levels observed in all groups were close to this normal range. Therefore, it can be concluded that the total protein values across all groups remained within the normal limits.

The results showed an increase in average blood glucose levels in mice from all groups, except for the acute control group of female mice. The average blood glucose levels in male BALB/c mice are reported to be 160.40 ± 26 mg/dL, while in female mice the levels are 144.9 ± 30.47 mg/dL [21]. Glucose levels in the blood may increase shortly after feeding [29]. Blood glucose is derived from dietary carbohydrates as well as glycogen stores in the liver and muscles. Several factors can contribute to elevated blood glucose levels, including the type of feed used. In this study, the mice were fed pellets rich in carbohydrates, which are rapidly metabolized into glucose. Another possible contributing factor is that blood samples may have been collected shortly after feeding, potentially leading to transient postprandial hyperglycemia.

Assessment of hepatic function is generally based on SGOT/AST (Serum Glutamic Oxaloacetic Transaminase/Aspartate aminotransferase) and SGPT/ALT (Serum Glutamic Pyruvic Transaminase/Alanine aminotransferase) enzyme levels in laboratory animals. Under normal physiological conditions, serum concentrations of SGOT and SGPT are typically low due to their intracellular localization. According to reference [21], the average normal AST level in male BALB/c mice is 135.20 ± 26.53 U/L, while in females it is 156.70 ± 57.20 U/L. Compared to these baseline values, increased SGOT levels were observed in the experimental. Therefore, histopathological evaluation remains essential to confirm the presence of structural liver tissue damage [30].

Damage to hepatic cells, as well as to other organs, tissues, and their constituent cells, can lead to elevated levels of SGOT in the bloodstream [31]. A substantial increase in serum SGOT levels—ranging from 10 to 100 times the normal range—may indicate severe hepatic injury, such as viral hepatitis or toxic hepatic necrosis, or may suggest cardiac cell damage [32]. Additionally, SGPT levels were found to be lower than the normal reference range; however, several sources suggest that this finding may not have significant clinical implications. This finding was correlated with the histopathological examination of the liver and heart, which showed no evidence of tissue damage.

Hematological and blood chemistry reference values may vary due to several factors, including age, sex, diet, housing conditions, physical activity, stress, reproductive status, and other physiological variables. In this study, all animals were carried out and tested in Animal BSL-3 Facilities. Therefore, in this study, the reference values were validated by comparison with control mice maintained under the same environmental and experimental conditions.

The interpretation of these values was also supported by histopathological analysis of multiple organs, including the bone marrow, brain, lungs, heart, thymus, liver, spleen, kidneys, lymphoid tissues, reproductive organs, and muscle. These organs displayed normal cellular and tissue structures. Histopathological alterations observed across all experimental groups included congestion (<20%), hemorrhage (<20%), necrosis (<5%), and degeneration (<15%).

Hydropic degeneration was noted in the liver and kidneys but remained within the mild range (<15%). This form of degeneration reflects cellular injury, characterized by intracellular fluid accumulation leading to cell swelling and the appearance of cytoplasmic vacuolation under microscopic examination. Despite these histological findings, all mice remained in good clinical condition, showing no signs of disease or mortality throughout the study.

This finding is consistent with the study by Thoresen (2024) [2], which reported that both in vitro and in vivo evaluations in non-human primates vaccinated with the tetravalent virus-like particle (VLP) vaccine demonstrated no evidence of enhanced Antibody-Dependent Enhancement (ADE) activity. Moreover, throughout the observation period, no clinical adverse events or signs of toxicity were observed in the vaccinated subjects, thereby indicating a favorable preliminary safety profile. Therefore, the tetravalent dengue VLP vaccine candidate demonstrates a strong safety profile in preclinical models, supporting its advancement to further long-term safety evaluations and eventual clinical trials in humans.

Previous studies have demonstrated that VLP-based vaccines are capable of efficiently stimulating dendritic cell activation, thereby initiating strong immune responses. Tariq et al. (2022) highlighted that VLPs represent revolutionary vaccine platforms with the capacity to activate antigen-presenting cells, including dendritic cells, both in vitro and in vivo across different infectious diseases [10]. Tang et al. (2012) reported that dengue virus type 1 VLPs produced in *Pichia pastoris* elicited virus-neutralizing antibodies and robust T cell responses in mice, underscoring the role of dendritic cell-mediated antigen presentation [12]. Similarly, Zhang et al. (2011) demonstrated that dengue VLP vaccination induced both humoral and cellular immune responses in BALB/c mice, supporting dendritic cell activation as a central mechanism bridging innate and adaptive immunity [13]. Collectively, these findings strengthen the rationale that dengue VLPs are highly immunogenic platforms capable of stimulating potent dendritic cell responses in both in vitro and in vivo models.

Although this study primarily assessed the toxicity profile of tetravalent dengue VLPs, these promising safety results provide a crucial foundation for advancing to immunogenicity studies, particularly focusing on dendritic cell activation mechanisms.

## 5. Conclusions

The data obtained in the present study demonstrate that administration of the tetravalent dengue virus-like particle (VLP) did not produce significant adverse effects on the physiological functions, clinical pathology, and histopathology of BALB/c mice. These findings from the preclinical study of the dengue VLP can be further explored in a clinical test in humans. These preclinical findings support the favorable safety profile of tetravalent dengue VLPs and provide a rationale for their progression to human clinical trials. Considering their previously reported ability to activate dendritic cells, further studies are warranted to evaluate their immunogenicity and potential role in dendritic cell-targeted immunotherapy.

### 5.1. Limitations

This study was conducted using a BALB/c mouse model, which, while providing valuable preclinical safety data, may not fully replicate the complex immune responses observed in humans. Additionally, the observation period was limited to 28 days, restricting the ability to assess potential long-term effects of the vaccine candidate. The use of a single animal species also limits the generalizability of the findings to other models or clinical settings. Furthermore, this study did not evaluate the immunogenicity or protective efficacy of the vaccine, which are important parameters to be addressed in subsequent research.

### 5.2. Future Research

Future studies should focus on evaluating the long-term safety of the tetravalent dengue VLP vaccine candidate. Additional animal models, including non-human primates, should be considered to provide more representative data for human applications. It is also recommended to conduct dose-optimization studies and extended observation periods to assess immune persistence and delayed adverse effects. Furthermore, investigations into T-cell responses against all four DENV serotypes, while minimizing the potential risk of Antibody-Dependent Enhancement (ADE), will be essential prior to initiating human clinical trials. Subsequent studies should also explore dendritic cell activation profiles following VLP administration, including cytokine secretion and antigen presentation capabilities, to validate the immunotherapeutic potential suggested by previous flavivirus VLP studies.

## Figures and Tables

**Table 1:** Hematological parameters of mice in the acute group.

Hematology Indices	Gender	Control Group (Mean ± SD)	VLP Group (Mean ± SD)	*p*-Value
Hematocrit (%)	Male	40.30 ± 1.06	39.98 ± 1.15	0.65
	Female	39.95 ± 0.60	35.86 ± 8.56	0.32
Hb (g/dL)	Male	12.40 ± 0.35	12.24 ± 0.29	0.45
	Female	14.35 ± 0.67	12.12 ± 3.03	0.15
RBC (10^6^/mm^3^)	Male	8.51 ± 0.43	8.34 ± 0.26	0.47
	Female	8.62 ± 0.27	8.07 ± 1.41	0.42
WBC/Leucocyte (10^3^/mm^3^)	Male	6.65 ± 1.67	6.67 ± 1.72	0.98
	Female	14.55 ± 1.31	10.70 ± 6.43	0.23
Thrombocyte (10^3^/mm^3^)	Male	613.00 ± 3.54	655.00 ± 165.68	0.58
	Female	545.50 ± 74.63	552.00 ± 103.73	0.91

Hematological parameters of male and female BALB/c mice in the acute toxicity study following dengue VLP administration compared to the control group.

**Table 2:** Hematological parameters of mice in the chronic group.

Hematology Indices	Gender	Control Group (Mean ± SD)	VLP Group (Mean ± SD)	*p*-Value
Hematocrit (%)	Male	33.63 ± 3.01	31.20 ± 4.46	0.3
	Female	37.43 ± 1.54	33.17 ± 5.23	0.12
Hb (g/dL)	Male	10.83 ± 1.11	10.32 ± 1.21	0.5
	Female	12.50 ± 0.75	11.20 ± 1.78	0.17
RBC (10^6^/mm^3^)	Male	7.76 ± 0.40	7.59 ± 0.61	0.6
	Female	8.21 ± 0.31	8.22 ± 0.67	0.98
WBC/Leucocyte (10^3^/mm^3^)	Male	7.77 ± 2.73	10.56 ± 5.57	0.34
	Female	7.13 ± 1.02	7.93 ± 0.60	0.17
Thrombocyte (10^3^/mm^3^)	Male	971.00 ± 294.77	889.67 ± 125.72	0.58
	Female	527.33 ± 111.41	702.00 ± 175.17	0.1

Hematological parameters of male and female BALB/c mice in the chronic toxicity study following dengue VLP administration compared to the control group.

**Table 3:** Blood biochemical parameters of mice in the acute group.

Biochemical Markers	Gender	Control Group (Mean ± SD)	VLP Group (Mean ± SD)	*p*-Value
Creatinine (mg/dL)	Male	0.47 ± 0.04	0.41 ± 0.15	0.42
	Female	0.46 ± 0.15	0.47 ± 0.15	0.87
BUN (mg/dL)	Male	17.80 ± 0.85	17.73 ± 0.97	0.9
	Female	17.10 ± 1.72	16.64 ± 0.83	0.6
Albumin/Globulin Ratio (g/dL)	Male	2.05 ± 0.24	1.90 ± 0.37	0.47
	Female	1.91 ± 0.24	2.08 ± 0.46	0.48
Albumin	Male	3.30 ± 0.07	3.18 ± 0.30	0.41
	Female	3.10 ± 0.14	3.04 ± 0.18	0.57
Globulin	Male	1.67 ± 0.23	1.53 ± 0.24	0.37
	Female	1.70 ± 0.29	1.51 ± 0.29	0.33
Total Protein (g/dL)	Male	4.97 ± 0.30	4.91 ± 0.39	0.8
	Female	4.80 ± 0.43	4.57 ± 0.33	0.37
Glucose (mg/dL)	Male	232.50 ± 8.84	215.75 ± 49.67	0.48
	Female	126.00 ± 36.77	127.80 ± 29.23	0.9
SGOT (U/L)	Male	216.50 ± 27.22	203.75 ± 50.17	0.72
	Female	262.50 ± 79.55	197.00 ± 30.12	0.12
SGPT (U/L)	Male	52.50 ± 5.30	53.75 ± 20.12	0.63
	Female	65.00 ± 3.54	53.00 ± 19.24	0.2

Blood biochemical parameters of male and female BALB/c mice in the acute toxicity study following dengue VLP administration compared to the control group.

**Table 4:** Blood biochemical parameters of mice in the chronic group.

Biochemical Markers	Gender	Control Group (Mean ± SD)	VLP Group (Mean ± SD)	*p*-Value
Creatinine (mg/dL)	Male	0.42 ± 0.07	0.45 ± 0.11	0.9
	Female	0.42 ± 0.08	0.43 ± 0.02	0.8
BUN (mg/dL)	Male	19.30 ± 0.54	18.70 ± 1.86	0.5
	Female	19.93 ± 0.54	20.83 ± 0.77	0.67
Albumin/Globulin Ratio (g/dL)	Male	1.89 ± 0.36	1.88 ± 0.39	0.3
	Female	1.74 ± 0.24	1.86 ± 0.02	0.3
Albumin	Male	3.10 ± 0.07	2.99 ± 0.14	0.16
	Female	2.97 ± 0.16	3.10 ± 0.19	0.27
Globulin	Male	1.70 ± 0.25	1.92 ± 0.16	0.14
	Female	1.73 ± 0.16	1.67 ± 0.11	0.49
Total Protein (g/dL)	Male	4.81 ± 0.18	4.92 ± 0.17	0.34
	Female	4.67 ± 0.01	4.76 ± 0.28	0.5
Glucose (mg/dL)	Male	297.33 ± 44.66	282.67 ± 44.18	0.61
	Female	263.67 ± 11.58	271.33 ± 14.51	0.38
SGOT (U/L)	Male	242.00 ± 51.40	202.00 ± 14.56	0.13
	Female	261.33 ± 41.21	248.00 ± 37.52	0.6
SGPT (U/L)	Male	40.67 ± 7.79	49.33 ± 4.55	0.06
	Female	96.00 ± 34.32	93.33 ± 49.40	0.9

Blood biochemical parameters of male and female BALB/c mice in the chronic toxicity study following dengue VLP administration compared to the control group.

## Data Availability

The data presented in this study are available on reasonable request from the corresponding author.

## Supplementary Materials

The following supporting information can be downloaded at: https://www.mdpi.com/article/10.3390/vaccines13111142/s1, Table S1: Mean body temperature of mice in the acute group; Table S2: Mean body temperature of mice in the chronic group; Table S3: Mean body weight of mice in the acute group; Table S4: Mean body weight of mice in the chronic group.

## Abbreviations

The following abbreviations are used in this manuscript:

DENV	Dengue Virus
VLP	Virus-Like Particle
ABSL-3	Animal Biosafety Level-3
BUN	Blood Urea Nitrogen
CBC	Complete Blood Count
SGPT	Serum Glutamic Pyruvic Transaminase
SGOT	Serum Glutamic Oxaloacetic Transaminase
AST	Aspartate Aminotransferase
ALT	Alanine Aminotransferase
Nab	Neutralizing Antibodies
ADE	Antibody-Dependent Enhancement
IACUC	Institutional Animal Care and Use Committee
WHO	World Health Organization
PBS	Phosphate-Buffered Saline
