# Supplementary material for: Targeting Dendritic Cells with Virus-like Particles: Toward Safer and More Immunogenic Vaccines
Source: Vaccines (Basel). 2025 Nov 6;13(11):1142. doi: 10.3390/vaccines13111142 (PMC12656481; doi:10.3390/vaccines13111142)
Supplement: Supplementary file 1 [file vaccines-13-01142-s001.zip › vaccines-3863323-supplementary.pdf]

vaccines

MDPI

3. Results

3.1. Body temperature

Body temperature in acute and chronic vaccine groups was in the same range as the control during the study period (Table S1 and Table S2).

Table S1. Mean body temperature of mice in the acute group

Observation day	Gender	Control group (Mean ± SD)	VLP group (Mean ± SD)
Day 0	Male	35.6 ± 0.68	35.9 ± 0.38
	Female	35.6 ± 0.35	36.0 ± 0.24
Day 1	Male	35.3 ± 0.44	35.65 ± 0.36
	Female	35.6 ± 0.54	35.80 ± 0.23
Day 2	Male	35.54 ± 0.54	35.64 ± 0.46
	Female	35.62 ± 0.33	35.73 ± 0.49
Day 3	Male	35.73 ± 0.51	35.74 ± 0.29
	Female	36.11 ± 0.11	36.10 ± 0.09
Day 4	Male	35.44 ± 0.63	35.70 ± 0.54
	Female	35.67 ± 0.35	35.57 ± 0.36
Day 7	Male	35.89 ± 0.12	35.40 ± 0.65
	Female	35.86 ± 0.11	35.95 ± 0.10
Day 14	Male	36.06 ± 0.18	35.86 ± 0.26
	Female	35.73 ± 0.62	35.99 ± 0.10

Mean body temperature (°C) of male and female BALB/c mice in the acute toxicity study following dengue VLP or control (PBS) administration at different observation days.

Table S2. Mean body temperature of mice in the chronic group

Observation day	Gender	Control group (Mean ± SD)	VLP group (Mean ± SD)
Day 0	Male	35.90 ± 0.69	36.04 ± 0.08
	Female	35.83 ± 0.38	35.53 ± 0.70
Day 1	Male	35.51 ± 0.63	35.63 ± 0.64
	Female	35.30 ± 0.74	35.55 ± 0.50
Day 2	Male	35.98 ± 0.23	35.74 ± 0.74
	Female	35.69 ± 0.45	35.46 ± 0.75
Day 3	Male	35.99 ± 0.16	35.77 ± 0.55
	Female	35.91 ± 0.49	36.03 ± 0.09
Day 4	Male	35.63 ± 0.58	35.46 ± 0.64
	Female	35.71 ± 0.79	35.70 ± 0.32
Day 7	Male	36.00 ± 0.28	35.87 ± 0.44
	Female	35.43 ± 1.16	36.03 ± 0.13
Day 14	Male	36.04 ± 0.11	35.76 ± 0.44
	Female	35.52 ± 1.01	35.96 ± 0.29

Mean body temperature (°C) of male and female BALB/c mice in the chronic toxicity study following dengue VLP or control (PBS) administration at different observation days.

3.2 Body weight and food consumption

The average weekly feed consumption of mice increased in first week, then decreased in 2nd week in all mice in acute treatment group (both males and females). In chronic group there was a decrease in feed consumption in 2nd week and 3rd week, then increased again in 4th week. The average body weight of animals treated with acute and chronic groups was in the same range as the control during the study period (Table S3 and Table S4).

Table S3. Mean body weight of mice in the acute group

Observation day	Gender	Control group (Mean ± SD)	VLP group (Mean ± SD)
Day 0	Male	28.5 ± 2.84	27.80 ± 2.66
	Female	25.60 ± 3.44	26.80 ± 4.66
Day 1	Male	28.00 ± 3.02	27.40 ± 2.88
	Female	25.60 ± 4.01	27.40 ± 4.74
Day 2	Male	28.50 ± 3.14	27.60 ± 2.63
	Female	25.60 ± 3.60	26.80 ± 4.89
Day 3	Male	29.00 ± 3.13	27.90 ± 2.51
	Female	25.20 ± 3.58	26.60 ± 4.97
Day 4	Male	29.20 ± 3.29	28.20 ± 2.49
	Female	25.70 ± 3.50	27.00 ± 5.01
Day 7	Male	29.40 ± 3.72	28.90 ± 1.91
	Female	26.60 ± 3.60	27.90 ± 5.00
Day 14	Male	31.00 ± 3.68	28.80 ± 4.39
	Female	26.10 ± 4.15	27.40 ± 4.67

Mean body weight (g) of male and female BALB/c mice in the acute toxicity study at various observation days after dengue VLP or control (PBS) administration.

Table S4. Mean body weight of mice in the chronic group

Observation day	Gender	Control group (Mean ± SD)	VLP group (Mean ± SD)
Day 0	Male	28.5 ± 2.84	27.80 ± 2.66
	Female	25.60 ± 3.44	26.80 ± 4.66
Day 1	Male	28.00 ± 3.02	27.40 ± 2.88
	Female	25.60 ± 4.01	27.40 ± 4.74
Day 2	Male	28.50 ± 3.14	27.60 ± 2.63
	Female	25.60 ± 3.60	26.80 ± 4.89
Day 3	Male	29.00 ± 3.13	27.90 ± 2.51
	Female	25.20 ± 3.58	26.60 ± 4.97
Day 4	Male	29.20 ± 3.29	28.20 ± 2.49
	Female	25.70 ± 3.50	27.00 ± 5.01
Day 7	Male	29.40 ± 3.72	28.90 ± 1.91
	Female	26.60 ± 3.60	27.90 ± 5.00
Day 14	Male	31.00 ± 3.68	28.80 ± 4.39

	Female	26.10 ± 4.15	27.40 ± 4.67

Mean body weight (g) of male and female BALB/c mice in the chronic toxicity study at various observation days after dengue VLP or control (PBS) administration.
